# Plasma Free Amino Acid Profiles Predict Four-Year Risk of Developing Diabetes, Metabolic Syndrome, Dyslipidemia, and Hypertension in Japanese Population

**DOI:** 10.1038/srep11918

**Published:** 2015-07-09

**Authors:** Minoru Yamakado, Kenji Nagao, Akira Imaizumi, Mizuki Tani, Akiko Toda, Takayuki Tanaka, Hiroko Jinzu, Hiroshi Miyano, Hiroshi Yamamoto, Takashi Daimon, Katsuhisa Horimoto, Yuko Ishizaka

**Affiliations:** 1 Center for Multiphasic Health Testing and Services, Mitsui Memorial Hospital, 1 Kanda, Izumicho, Chiyoda-ku, Tokyo 101-8643, Japan; 2 Institute for Innovation, Ajinomoto Co., Inc., 1-1 Suzuki-cho, Kawasaki-ku, Kawasaki 210-8681, Japan; 3 Department of Biostatistics, Hyogo College of Medicine, 1-1, Mukogawa-cho, Nishinomiya, Japan; 4 Molecular Profiling Research Center for Drug Discovery, National Institute of Advanced Industrial Science and Technology, Tokyo, Japan

## Abstract

Plasma free amino acid (PFAA) profile is highlighted in its association with visceral obesity and hyperinsulinemia, and future diabetes. Indeed PFAA profiling potentially can evaluate individuals’ future risks of developing lifestyle-related diseases, in addition to diabetes. However, few studies have been performed especially in Asian populations, about the optimal combination of PFAAs for evaluating health risks. We quantified PFAA levels in 3,701 Japanese subjects, and determined visceral fat area (VFA) and two-hour post-challenge insulin (Ins120 min) values in 865 and 1,160 subjects, respectively. Then, models between PFAA levels and the VFA or Ins120 min values were constructed by multiple linear regression analysis with variable selection. Finally, a cohort study of 2,984 subjects to examine capabilities of the obtained models for predicting four-year risk of developing new-onset lifestyle-related diseases was conducted. The correlation coefficients of the obtained PFAA models against VFA or Ins120 min were higher than single PFAA level. Our models work well for future risk prediction. Even after adjusting for commonly accepted multiple risk factors, these models can predict future development of diabetes, metabolic syndrome, and dyslipidemia. PFAA profiles confer independent and differing contributions to increasing the lifestyle-related disease risks in addition to the currently known factors in a general Japanese population.

The obesity and diabetes epidemic is spreading to parts of the world including Asian regions where historically the problem has been less pronounced, and the rates of increase show no signs of slowing^1^. While interethnic differences between Asian and Western populations regarding pathophysiology of the obesity and diabetes are well known^2^, visceral fat accumulation and insulin resistance are one of the key factors for developing metabolic diseases. While both genetic factors such as defects in the insulin signaling pathway^3^,^4^, and environmental factors jointly affect the susceptibility^5^, the environmental factors of global shift toward the western lifestyle of eating and sedentary habits play pivotal roles in the rising prevalence of these diseases^6^.

In the post-genomic era, the development of analytical techniques and instruments improve the metabolic assessment of physiological states, which is useful for deeply understanding the mechanisms underlying disease etiology and more preventive healthcare approaches^7^. Metabolic disturbances that are associated with visceral obesity and hyperinsulinemia are thought to be important in the progression of lifestyle-related diseases^8^,^9^, and the early detection of these disturbances is important for both the prevention of diseases and reducing medical costs.

Some studies revealed that the levels of plasma free amino acids (PFAAs), especially branched-chain amino acids (BCAAs) and aromatic amino acids (AAAs), were associated with visceral obesity, insulin resistance, and diabetes mellitus (DM) in several cross-sectional and prospective cohort studies^10^,^11^,^12^,^13^,^14^,^15^,^16^,^17^. Indeed, a specific role of intra-abdominal fat accumulation is indicated in the relationship between visceral obesity and metabolic dysfunction^8^,^9^. Along with this context, we recently showed that several PFAAs, including BCAAs, AAAs, alanine (Ala), glycine (Gly), and proline (Pro), were specifically correlated with visceral and not subcutaneous fat deposition independent of body mass index (BMI) in a study that used computed tomography (CT) imaging^10^. Additionally, while after controlling for BMI, commonly accepted risk factors for insulin resistance, including circulating fatty acids and inflammatory cytokines, did not affect insulin dysregulation^12^, insulin resistance was shown to be correlated with the alterations of PFAA levels, such as BCAAs, AAAs, and a cluster of BCAAs and related amino acids^12^. These findings underscore the potential central role of the shift of amino acid metabolism early in the pathogenesis of lifestyle-related diseases, but few relevant studies have been conducted for examining the optimal combination of PFAAs to evaluate health risks, especially in Asian populations. Furthermore, though the evaluation of individuals’ future risks of developing lifestyle-related diseases other than diabetes may be enabled through the assessment of PFAA levels, few relevant studies have been performed.

Recently we developed, novel, multivariate regression model of PFAAs, which is referred to as “AminoIndex technology”, and utilized to compresses the multidimensional information from PFAA profiles into a single score to maximize the differences between case and control populations. By using this technology, we successfully distinguished between various disease states in rat models of type 1 and type 2 diabetes^18^, to determine the progression of liver fibrosis in chronic hepatitis C cases in humans^19^, to screen for several types of cancers^20^,^21^, and to distinguish visceral obese subjects^10^,^22^.

Here, we quantified PFAA levels in 865 subjects who had undergone evaluations of their visceral fat area (VFA) by CT scan, and in 1,160 subjects who had each been subjected to an oral glucose tolerance test (OGTT) to determine their 2-h post-challenge glucose and insulin levels (Glc120 min and Ins120min). For the above subjects, we performed the multiple linear regression analysis with variable selection to model the relationships between the PFAA profiles with the VFA or Ins120 min values, and even to compress multidimensional information on PFAAs into a single score, which is called as the PFAA index. Furthermore, we performed a cohort study to evaluate whether each PFAA index and single PFAA level could be used to predict the four-year risk of developing DM, metabolic syndrome, dyslipidemia, or hypertension in 2,984 general Japanese subjects with covariate adjustments.

## Results

### The alteration of PFAA profiles according to metabolic status was first confirmed in the large Japanese population

**Figure 1:**
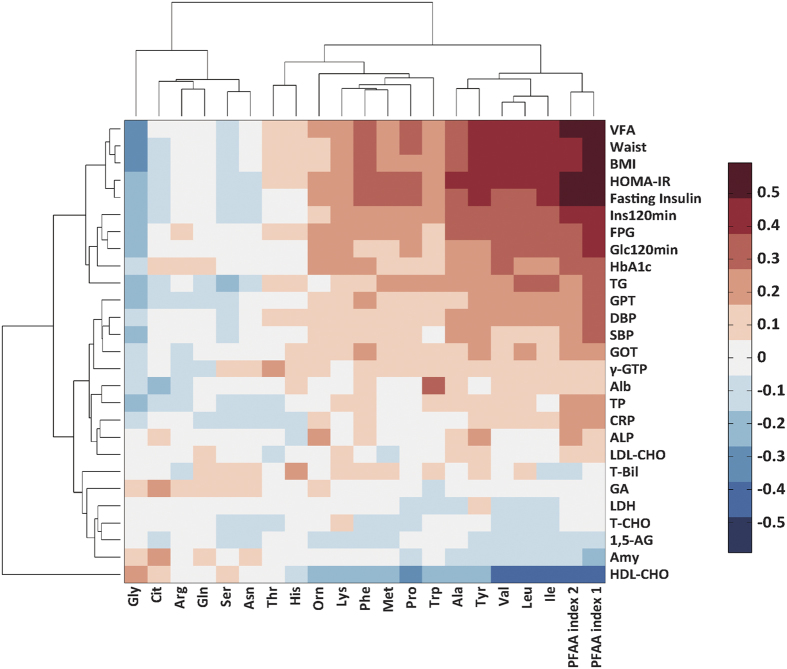
Correlation clustering using PFAA profiles and metabolic variables. Pearson’s correlation coefficients were calculated, and hierarchical clustering was conducted. HbA1c: Hemoglobin A1c, FPG: Fasting plasma glucose, Glc120 min: Plasma glucose level 2-h after OGTT, Ins120 min: Serum insulin level 2-h after OGTT, HOMA-IR: Homeostasis model assessment of insulin resistance, VFA: Visceral fat area by computed tomography, BMI: Body mass index, TG: Triglyceride, GPT: Alanine aminotransferase, DBP: Diastolic blood pressure, SBP: Systolic blood pressure, GOT: Aspartate aminotransferase, γ-GTP: gamma-Glutamyl transpeptidase, Alb: Albumin, TP: Total protein, T-Bil: Total bilirubin, LDL-CHO: LDL cholesterol, CRP: C-reactive protein, ALP: Alkaline phosphatase, GA: Glycoalbumin, Amy: Amylase, LDH: Lactate dehydrogenase, T-CHO: Total cholesterol, 1,5-AG: 1,5-anhydro-D-glucitol, HDL-CHO: HDL cholesterol.

We first examined whether PFAA profiles were altered in accordance with their metabolic status in the large Japanese population (Fig. 1). A correlation structure was depicted with an aid of hierarchical clustering, between the PFAA profiles and the metabolic variables that were associated with the lifestyle-related diseases. As easily seen in the figure, five amino acids (isoleucine (Ile), leucine (Leu), valine (Val), tyrosine (Tyr), and Ala) showed strong positive correlations with visceral obesity-, glucose-, and insulin-related variables, and only Gly showed negative correlations with them. Both the blood glucose levels and insulin levels belonged to the same cluster, which also contained obesity-related variables, including BMI, waist circumference, and VFA. This finding was consistent with the fact that both hyperinsulinemia and hyperglycemia were closely associated with visceral obesity. Unexpectedly, 1,5-anhydro-D-glucitol (1,5-AG) and glycoalbumin (GA), which are known to reflect short-term impaired blood glucose regulation, were only marginally correlated with the PFAA profiles. The glycemic changes that occurred over the course of several days may have had little effect on the PFAA levels. Also, the whole PFAA concentrations in the category of healthy subjects, patients of DM, metabolic syndrome, dyslipidemia, or hypertension at the beginning of the cohort study can be found as Supplementary Table S1 online.

**Figure 2:**
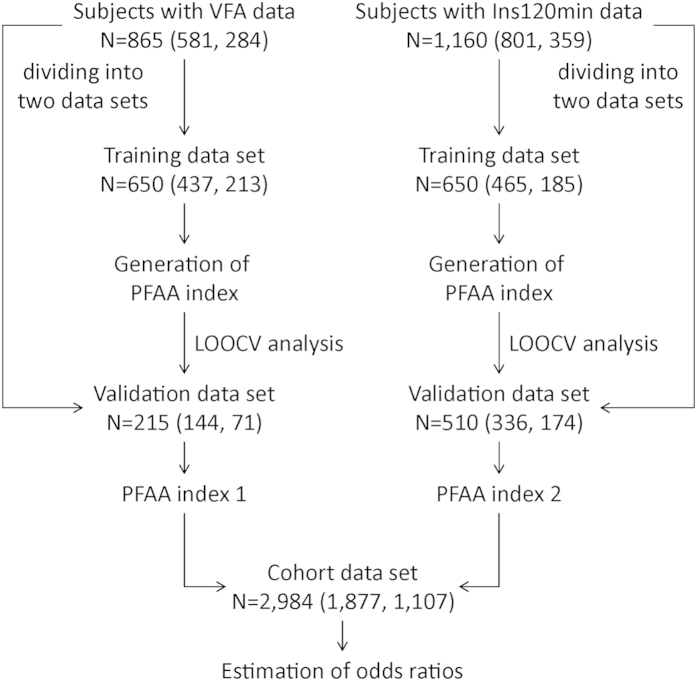
A schematic workflow of the study. The PFAA indices were generated by the subjects with VFA data or with Ins120 min data. The number of subjects (male, female) used for the model construction and validation is shown. PFAA index: the index generated by plasma free amino acid (PFAA) profiles, VFA: Visceral fat area by computed tomography, Ins120 min: Serum insulin level 2-h after OGTT, LOOCV: leave-one-out cross-validation.

Next, we had constructed the robust PFAA models, being validated by leave-one-out cross-validation (LOOCV) analysis and independent validation data set (Table 1 and Fig. 2). Supplementary Figure S1 depicts the frequencies of the kinds of PFAAs that were selected for the top 100 models. Regarding the PFAA index for VFA, almost all the models included Tyr, Ala, and Gly, whereas Ile and Tyr were included in the PFAA index for Ins120 min in almost all cases. These differences may be derived from the specific statuses of visceral fat deposition and postprandial hyperinsulinemia. In terms of Akaike’s Information Criterion (AIC), PFAA index 1, consisting of Leu, Ala, Tyr, asparagine (Asn), tryptophan (Trp), and Gly, was chosen to be the best model for VFA as an objective variable, and PFAA index 2, consisting of Ile, Ala, Tyr, phenylalanine (Phe), methionine (Met), and histidine (His), was the best model for Ins120 min as an objective variable. Because the obtained indices were potentially over-fitted, LOOCV analysis was performed to avoid the optimistic biased assessment. Then, the validation data set, which was divided in advance, was used to confirm the robustness of the obtained models. The correlation coefficient of PFAA index 1 against VFA in the training data set (r = 0.58, p < 0.0001) were replicated in the LOOCV analysis and validation data set (r = 0.62, p < 0.0001). Additionally, the correlation coefficient of PFAA index 2 against Ins120 min in the training data set (r = 0.47, p < 0.0001) were replicated in the LOOCV analysis and validation data set (r = 0.43, p < 0.0001). These correlation coefficients were higher compared with those of the single amino acids (Fig. 1). The correlation coefficient between PFAA index 1 and PFAA index 2 was 0.77 (p < 0.0001).

### The PFAA indices could be predictors for developing new-onset DM, metabolic syndrome, dyslipidemia, and hypertension within four years

To confirm the capabilities of the PFAA indices to predict future risk of developing DM, metabolic syndrome, dyslipidemia, or hypertension, we performed a cohort analysis (Table 2). The cohort analysis was carried out, in which the subjects without these diseases were selected from a total of 2,984 subjects (2,729 subjects without DM, 91.5%; 2,695 subjects without metabolic syndrome, 90.3%; 2,336 without dyslipidemia, 78.3%; or 2,637 without hypertension, 88.4%). These subjects were followed from 2008 until 2012 to determine who developed the diseases within the four-year period. Accordingly, 174 subjects, 300 subjects, 504 subjects, and 424 subjects presented with newly developed DM, metabolic syndrome, dyslipidemia, and hypertension, respectively. The basal PFAA concentrations were compared according to whether the subjects newly developed respective disease or not within the four year follow-up period in Table 2.

According to the above procedure, we successfully illustrated the PFAA indices capabilities in the cohort study. Indeed, the odds ratios of four-year development of DM, metabolic syndrome, dyslipidemia, or hypertension per 1 SD increment in PFAA index 1 were 2.06 (95% confidence interval: 1.70–2.50; p < 0.001), 3.04 (2.57–3.63; p < 0.001), 1.98 (1.74–2.26; p < 0.001), and 1.42 (1.25–1.61; p < 0.001), respectively (Table 3). These findings suggest that each increase of 1 SD for PFAA index 1 was related to an approximately 106% (70–150%) increased risk of the future development of DM, 204% (157–263%) increased risk of developing metabolic syndrome, 98% (74–126%) increased risk of developing dyslipidemia, and 42% (25–61%) increased risk of developing hypertension after adjusting for age and gender. Furthermore, each 1 SD increase in PFAA index 2 was related to an approximately 77% (50–110%) increased risk of the future development of DM, 127% (96–164%) increased risk of developing metabolic syndrome, 74% (54–97%) increased risk of developing dyslipidemia, and 31% (17–47%) increased risk of developing hypertension. Quartile analyses revealed a linear increase in risk of developing these diseases. In the top vs. bottom quartile of PFAA index 1 or 2, an approximately 7-fold or 5-fold increase in the risk of developing DM, 20-fold or 15-fold increases in the risk of metabolic syndrome, 5-fold or 4-fold increases in the risk of dyslipidemia, and a 2-fold increase in the risk of hypertension were observed.

### The prediction of four-year development of DM, metabolic syndrome and dyslipidemia after adjusting for additional covariates including HOMA-IR

Furthermore, we confirmed the versatile predictive capability for DM, metabolic syndrome, and dyslipidemia development by the PFAA indices, with adjustment of additional covariates including homeostasis model assessment of insulin resistance (HOMA-IR) (Table 4). For single PFAA levels, Ile, Leu, Tyr, and Phe were statistically significantly related to the development of DM over the four-year time period after adjusting for age, gender, BMI, fasting plasma glucose (FPG), and HOMA-IR. Ile, Leu, Tyr, Ala, and serine (Ser) were significantly related to the development of metabolic syndrome, and Ile, Leu, Val, Tyr, Ala, Pro, Ser, and Gly were significantly related to the development of dyslipidemia. Each 1 SD increase in PFAA index 1 or index 2 was associated with an approximately 31% (2–69%) or 39% (12–74%) increased risk of the future development of DM, 59% (29–96%) or 36% (14–63%) increased risk of developing metabolic syndrome, and 72% (47–102%) or 47% (28–69%) increased risk of developing dyslipidemia, respectively. No significant associations were found between either PFAA index and future hypertension development after adjusting for age, gender, BMI, FPG, and HOMA-IR.

### The PFAA indices confer independent and differing contributions to increasing DM, metabolic syndrome, and dyslipidemia risks in addition to the currently known risk factors

Even after adjusting for commonly accepted risk factors, we further confirmed that the PFAA indices could predict four-year risk of developing DM, metabolic syndrome, and dyslipidemia (Table 5). The adjustments for the following covariates were done: age, gender, BMI, FPG, HOMA-IR, waist circumference, HDL cholesterol (HDL-C), LDL cholesterol (LDL-C), total cholesterol (T-CHO), triglycerides (TG), systolic blood pressure (SBP), and diastolic blood pressure (DBP). Even after adjusting for all of the aforementioned variables, a 41% (12–76%) increased risk of developing metabolic syndrome and 34% (12–60%) increased risk of developing dyslipidemia were observed per 1 SD increase for PFAA index 1. For PFAA index 2, a 30% (4–63%) increased risk of developing DM, 26% (4–53%) increased risk of developing metabolic syndrome, and 20% (2–40%) increased risk of developing dyslipidemia per 1 SD increase were observed.

## Discussion

We first represented the evidence of large Japanese population that the indices generated from PFAA profiles are useful for identifying individuals who are at high risk in terms of visceral obesity or insulin dysregulation, which have previously been difficult to identify using conventional variables, such as BMI, FPG, or Hemoglobin A1c (HbA1c). The longitudinal analysis revealed that these PFAA indices and a few single PFAA levels could identify the subjects who are at higher risks of developing new-onset DM, metabolic syndrome, dyslipidemia, or hypertension over a four-year period. Early intervention for individuals who are at higher risks of developing lifestyle-related diseases is particularly important in terms of preventive medicine, particularly for the introduction of lifestyle modifications. Because macrovascular complications of the insulin-resistant state precede the onset of hyperglycemia and actually begin during the pre-diabetic phase^23^,^24^, this condition must be addressed even if the FPG or HbA1c levels are lower than the established clinical criteria.

Recently, the delineation of PFAA profiles in association with a variety of metabolites has become a prominent research focus. For example, 20 metabolites, including BCAAs and AAAs, were identified to be significantly associated with HOMA-IR values in 7,098 Finns^15^. A further longitudinal analysis was performed to investigate whether metabolite profiles could predict the development of DM in the Framingham Offspring Study over a 12-year period, which revealed that BCAA and AAA levels are significantly related to a future diagnosis of DM^14^. It was then further confirmed that these BCAAs and AAAs are novel markers of cardiovascular disease development and are early links between diabetes and cardiovascular disease susceptibility^25^. While the interethnic differences of pathophysiology of insulin dysregulation and visceral obesity between Asian and Western populations are well known^2^, very limited study has examined the relationship between PFAA profiles and lifestyle-related disease risks in Asian populations. The present study has confirmed that PFAA profiles can predict future DM development in the Japanese population, even within a relatively short period (four years). Additionally, these profiles can predict the future development of metabolic syndrome and dyslipidemia even after adjusting for commonly accepted risk factors, including age, gender, BMI, blood glucose, blood lipids, blood pressure, and HOMA-IR. PFAA index 1, which is a combination of PFAA profiles which can evaluate VFA, was more sensitive for detecting the future development of metabolic syndrome and dyslipidemia compared with the single amino acid levels. These results indicate that whereas PFAA profiles and additional variables involving insulin resistance, DM, metabolic syndrome, dyslipidemia, and hypertension are associated with each other, they are not synonymous. The PFAA profiles make independent and differing contributions to increasing lifestyle-related disease risks. Intriguingly, although the development of hypertension was significantly related to PFAA profiles after adjusting for age and gender, a significant relation was no longer observed after adjusting for the additional covariates, suggesting that each lifestyle-related disease has a different relationship with amino acid metabolism. The relationship between hypertension and amino acid metabolism could be more distant compared with that between DM, metabolic syndrome, and dyslipidemia, and amino acid metabolism. In fact, Fig. 1 shows that SBP and DBP belong to the different cluster from insulin- and obese-related variables in view of PFAA profiling. Comparing PFAA index 1 and 2, PFAA index 1 which correlates with VFA was more sensitive for predicting the future development of DM, metabolic syndrome, dyslipidemia, and hypertension (Table 3). Although, both visceral obesity and hyperinsulinemia are important in the development of lifestyle-related diseases^8^,^9^, these result could imply that visceral fat tissue play a critical role in the disease progression, and PFAA index 1 could be more versatile for future disease prediction.

Although the PFAA alterations are thought to be the appearance of metabolic shift along with the increasing risks of developing lifestyle-related diseases, different underlying mechanisms may exist for each type of PFAA level. Figure 1 depicts the cluster that is formed by Ile, Leu, Val, and Tyr, which is positively correlated with VFA and Ins120min. We previously reported that PFAA profiles, especially BCAAs and AAAs, specifically correlate with visceral but not subcutaneous fat deposition^10^. The elevation in blood BCAA levels was thought to be due to the reduced uptake of BCAAs into muscles, which was caused by decreased insulin activity and the decreased utilization of amino acids in muscles^26^. In addition, the abundance and/or activity of branched-chain alpha-keto acid dehydrogenase, which is a rate-limiting enzyme in BCAA oxidative catabolism in the liver and visceral adipose tissue, was found to be significantly reduced in both human subjects with insulin resistance and insulin-resistant rodent models relative to lean controls^27^,^28^. Actually, the blood concentrations of BCAAs and metabolites that are derived from partial BCAA catabolism were found to be elevated in cases of obesity or insulin resistance^29^,^30^. Although little is known about the mechanisms that elevate AAAs, it has been hypothesized that tyrosine aminotransferase is repressed during states of insulin resistance and DM, resulting in elevations of circulating Tyr and Phe^27^. In the current study, Gly, Ser, glutamine (Gln), and Asn formed a cluster that was negatively correlated with VFA, BMI, HOMA-IR, and Ins120 min (Fig. 1). In the Framingham Heart Study^31^, a high plasma ratio of Gln-to-Glu was associated with a lower incidence of DM, which is consistent with the present results. Because Gly and Ser share large portions of their metabolic pathways, the modifications of these pathways could reduce their levels. The reductions of these amino acids could be due to their enhanced utilizations due to accelerated gluconeogenesis, since glucose production from both Gly and Ser in hepatocytes is increased in diabetic individuals, whereas this type of glucose production is low in healthy conditions^32^. In fact, circulating levels of additional gluconeogenesis precursors, such as Ala, pyruvate, and lactate, have been reported to increase, which reflects impaired insulin sensitivity prior to the elevation of fasting glucose and insulin levels^15^,^16^,^17^. The set point of gluconeogenesis may differ in these populations, which may affect gluconeogenesis-related amino acid levels, such as Ala, Gln, Gly, and Ser. By improving the insulin-resistant state through weight loss by dietary intervention or gastric bypass surgery, these PFAA alterations are reported to be normalized^33^,^34^. In the experiment using adiponectin knockout mice, adiponectin administration corrected high-fat diet-induced metabolomic disturbances in muscle tissues, including those involving amino acid^35^. This suggests that PFAA is the marker that directly reflects metabolic status, and such metabolic disturbances may be directly linked to the development of DM, metabolic syndrome, and dyslipidemia.

Our study provides evidence for the importance of PFAAs as markers of metabolic aggravation; however, it is important to note that our results do not indicate that the ingestion of dietary protein or amino acids accelerates the aggravation of metabolic syndrome or the onset of DM. By contrast, many papers report that the dietary ingestion of BCAAs is responsible for some of the beneficial effects of high-protein diets^36^,^37^,^38^, including the amelioration of insulin resistance by Leu^39^, the hypoglycemic effect that is related to Ile^40^, and the amelioration of hepatic *de novo* lipogenesis and prevention of hepatic steatosis^41^,^42^. Additionally, human population-based studies on dietary BCAA intake and body weight have shown that higher levels of dietary BCAA intake are associated with lower prevalences of overweight and obese individuals^37^. Furthermore, a higher dietary intake of BCAAs has been reported to be related to a decreased risk of diabetes in the Japanese population in the Takayama study^43^. Therefore, the elevations of plasma BCAA level in the subjects who were at high risk for lifestyle-related diseases may have been paradoxical^28^, and further studies should be performed regarding the relationship between PFAA alterations and dietary ingestion.

In conclusion, our study underscores the importance of clinically evaluating PFAA profiles for assessing visceral obesity and hyperinsulinemia. The PFAA models were able to predict the four-year risk of developing lifestyle-related diseases, including DM, metabolic syndrome, dyslipidemia, and hypertension, in a general Japanese population, suggesting the usefulness of these indices as versatile markers for health monitoring.

## Methods

### Subjects and workflow

The study was conducted in accordance with the Declaration of Helsinki, and the protocol was approved by the Ethical Committees of Mitsui Memorial Hospital. A total of 3,701 Japanese subjects who had undergone the Ningen Dock comprehensive medical check-up system^44^ in 2008 at the Center for Multiphasic Health Testing and Services, Mitsui Memorial Hospital, were enrolled. The main inclusion criteria were as follows; subjects who had undergone the Ningen Dock comprehensive medical check-up system for periodic health examination, not taking antidiabetic medications regularly, aged at least 20 years, and giving informed consent to participate in the study. The patients with hepatitis C were excluded.

A schematic workflow of the study is illustrated in Fig. 2. The subject characteristics at baseline examination are shown in Table 1. Of these subjects, VFA and Ins120 min data were available for 865 and 1,160 subjects, respectively, for the development of amino acid indices. These data were divided into two data sets in chronological order, including training and validation data sets. For the cohort analysis, 2,984 subjects were continuously followed from 2008 to 2012, and newly developed cases of DM, metabolic syndrome, dyslipidemia, and hypertension were recorded.

### Measurement of metabolic variables and quantification of PFAAs

Blood samples were taken from the subjects after an overnight fast. Serum levels of T-CHO, HDL-C, LDL-C, TG, GA, and 1,5-AG^45^,^46^ were determined enzymatically. FPG was measured with the hexokinase method, and HbA1c was determined using the latex agglutination immunoassay. Serum insulin levels were measured immunologically, and HOMA-IR was calculated^47^. The VFA calculated from a CT scan at the level of the umbilicus were measured in 865 subjects, using FatScan software (N2 System Co., Osaka, Japan). All CT scans were performed in the supine posture at the umbilical level using a CT scanner (SOMATOM Sensation Cardiac 64, Siemens, Germany) based on the Japanese guidelines for obesity treatment by the Japan Society for the Study of Obesity^10^. A 75 g OGTT was performed, and Glc120 min and Ins120 min values were obtained for 1,160 subjects. The measurements of all other variables were performed as previously described^10^,^22^.

For the amino acid analyses, blood samples (5 mL) were collected from forearm veins after overnight fasting into tubes containing disodium ethylenediaminetetraacetate that were immediately placed on ice. The plasma amino acid concentrations were measured by high-performance liquid chromatography–electrospray ionization mass spectrometry followed by precolumn derivatization as previously described^10^,^20^,^48^,^49^. The following 19 amino acids were measured: Ala, arginine (Arg), Asn, citrulline (Cit), Gln, Gly, His, Ile, Leu, lysine (Lys), Met, ornithine (Orn), Phe, Pro, Ser, threonine (Thr), Trp, Tyr, and Val.

### Clinical assessment

Metabolic syndrome was defined according to the Japanese diagnostic criteria for metabolic syndrome. Subjects with metabolic syndrome had visceral obesity (waist ≥ 85 cm in males and ≥90 cm in females) plus at least 2 of the following three components: (1) HDL-C < 40 mg/dL, TG ≥ 150 mg/dL, or the use of medication for dyslipidemia; (2) FPG ≥ 110 mg/dL or the use of medication for diabetes; and (3) blood pressure ≥130/85 mmHg or the use of antihypertensive medication. Diabetes was defined in patients with FPG ≥126 mg/dL, HbA1c ≥ 6.5%, or those who were taking medication for diabetes. Dyslipidemia was defined in individuals with fasting LDL-C ≥ 140 mg/dL, HDL-C < 40 mg/dL, TG ≥ 150 mg/dL, or those who were taking medication for dyslipidemia. Hypertension was defined in patients with blood pressure ≥140/90 mmHg or those who were taking antihypertensive medications.

### Construction of PFAA indices

Using the training data set, multiple linear regression analysis with variable selection was performed to model the relationships between the PFAA levels (independent variables) and the VFA or Ins120 min values (dependent variable)^7^,^10^,^20^. The number of PFAAs in the multiple linear regression model were restricted to six and fewer among all of the possible combinations of PFAAs. The top 100 best-fit models were chosen with regard to AIC. Finally, among these models, two models were focused on, which yielded the best fit for VFA and Ins120 min, respectively. The estimating equations obtained from the two models, which enabled us to compress multidimensional information on the PFAA levels into a single score, were called as the PFAA index 1 and index 2.

To address the over-fitting potential of each model, a LOOCV analysis was performed. Briefly, in the LOOCV analysis, one sample was omitted from the training data set, the candidate model was fitted to the remaining samples, and the value of the PFAA index for the left-out sample was calculated from the fitted model. This process was repeated until every sample in the training data set had been left out once. Validation was also performed using the validation data set, which was prepared in advance.

### Correlation between PFAAs and metabolic variables

Correlation analysis between each of single PFAA levels and PFAA indices and metabolic variables was performed using the Pearson product-moment correlation coefficient (in 834 subjects in which the VFA, Ins120 min, 1,5-AG, and GA values and additional data were available). In addition, two-dimensional hierarchical cluster analysis that was based on the correlation coefficient matrix between the PFAA levels and additional measured variables was performed.

### Cohort analysis of four-year risk of developing DM, metabolic syndrome, dyslipidemia, or hypertension

To determine whether each PFAA index and single PFAA level were related to the four-year risk of developing DM, metabolic syndrome, dyslipidemia, and hypertension, we examined the relationships of the PFAA profiles to these lifestyle-related diseases in a cohort of 2,984 subjects. To test for the risk of developing DM, metabolic syndrome, dyslipidemia, or hypertension, the subjects without these diseases were selected and followed from 2008 until 2012 to determine who developed the diseases during the four years. More specifically, from 2,984 subjects in the cohort study, the subjects without DM (2,729 subjects), the subjects without metabolic syndrome (2,695 subjects), the subjects without dyslipidemia (2,336 subjects), and the subjects without hypertension (2,637 subjects) were included in the follow-up study, respectively. The basal PFAA concentrations were compared according to whether the subjects newly developed respective disease or not within the four year follow-up period (Table 2).

The PFAA indices and all of the amino acids were scaled to multiples of 1 SD. A logistic regression analysis was used to assess the contribution of each PFAA index and single PFAA level in the development of these diseases over the four-year period. For the PFAA indices and their quartiles, two different models were used as follows: 1) those adjusting for age and gender and 2) those adjusting for age and gender in addition to BMI, FPG, and HOMA-IR as continuous variables. For a single PFAA concentration, a logistic regression analysis using each single PFAA as a continuous variable was performed, adjusting for age, gender, BMI, FPG, and HOMA-IR. The information on the medication therapies such as lipid- or glucose- lowering drugs was not taken into consideration in the analysis, because patients who regularly take medications in the first year were not included in the study. More detailed analyses of DM, metabolic syndrome, and dyslipidemia, in which PFAA indices showed comparatively higher performances were conducted with further adjustments using the following covariates: age, gender, BMI, FPG, HOMA-IR, waist circumference, HDL-C, LDL-C, T-CHO, TG, SBP, and DBP.

All of the probability values were two-sided, and values corresponding with p < 0.05 were considered to be statistically significant. MATLAB (The Mathworks, Natick, MA, USA), GraphPad Prism (GraphPad Software, La Jolla, CA, USA), and JMP 9.0 (SAS Institute Inc., Cary, NC, USA) were used for the statistical analyses. All of the data were analyzed anonymously throughout the study.

## Additional Information

**How to cite this article**: Yamakado, M. *et al.* Plasma Free Amino Acid Profiles Predict Four-Year Risk of Developing Diabetes, Metabolic Syndrome, Dyslipidemia, and Hypertension in Japanese Population. *Sci. Rep.* **5**, 11918; doi: 10.1038/srep11918 (2015).

## Supplementary Material

Supplementary Table S1

Supplementary Figure S1

## Figures and Tables

**Table 1:** Demographic and clinical characteristics.

	Construction of PFAA index for VFA		Construction of PFAA index for Ins120 min		Cohort study
	Training data	Validation data	Training data	Validation data	
N (male, female)	(437, 213)	(144, 71)	(465, 185)	(336, 174)	(1877, 1107)
Age	61.1 ± 11.5	60.6 ± 10.9	57.4 ± 11.3	60.3 ± 10.7	55.2 ± 10.1
BMI	23.4 ± 3.1	23.7 ± 3.7	23.4 ± 3.1	23.5 ± 3.4	22.8 ± 3.3
Waist (cm)	84.3 ± 9.0	85.1 ± 9.5	83.8 ± 8.7	84.8 ± 9.1	83.0 ± 9.4
FPG (mg/dL)	100.3 ± 21.1	100.8 ± 18.4	98.7 ± 15.8	98.1 ± 15.9	98.1 ± 16.6
HbA1c (%)	5.8 ± 0.6	5.9 ± 0.7	5.8 ± 0.5	5.8 ± 0.5	5.7 ± 0.6
HOMA-IR	1.9 ± 1.4	2.1 ± 1.7	1.8 ± 1.2	1.9 ± 1.3	1.6 ± 1.2
Systolic Blood Pressure (mmHg)	125.3 ± 17.7	128.4 ± 19.1	127.3 ± 18.2	124.9 ± 17.5	122.3 ± 18.2
Diastolic Blood Pressure (mmHg)	78.5 ± 11.0	80.5 ± 11.4	80.0 ± 11.2	78.4 ± 10.8	77.3 ± 11.1
Triglycerides (mg/dL)	125.7 ± 117.6	120.9 ± 74.8	121.4 ± 114.7	123.8 ± 80.8	113.1 ± 130.8
Total Cholesterol (mg/dL)	207.2 ± 31.6	211.2 ± 32.1	207.2 ± 32.5	210.0 ± 31.6	207.1 ± 33.3
HDL Cholesterol (mg/dL)	60.1 ± 15.3	60.8 ± 15.8	60.3 ± 14.8	59.8 ± 15.4	61.1 ± 15.4
LDL Cholesterol (mg/dL)	122.9 ± 30.2	125.5 ± 29.6	125.2 ± 30.8	124.8 ± 29.7	124.3 ± 29.6
Visceral fat area (cm^2^)	120.7 ± 60.8	141.4 ± 67.1	—	—	—
Ins120min (μU/mL)	46.6 ± 38.5	50.0 ± 50.4	41.9 ± 31.2	49.6 ± 46.2	—
Metabolic syndrome (%)	15.8	18.6	16.6	15.3	9.7
DM (%)	14.8	18.1	11.1	13.9	8.5
Dyslipidemia (%)	24.6	25.1	24.6	25.1	21.7
Hypertension (%)	23.1	34.0	28.0	22.7	11.6

The continuous variables are summarized as means ± standard deviations (SD) and the categorical variables as frequencies and proportions.

**Table 2:** Baseline plasma free amino acid concentrations in the cohort study.

	DM		Metabolic syndrome		Dyslipidemia		Hypertension	
	Non-developed	Developed	Non-developed	Developed	Non-developed	Developed	Non-developed	Developed
N(male, female)	2555(1520, 1035)	174(146, 28)	2395(1324, 1071)	300(278, 22)	1832(928, 904)	504(377, 127)	2213(1271, 942)	424(335, 89)
Gly	210.9 ± 50.5	195.4 ± 44.4***	213.2 ± 51.6	189.5 ± 32.8***	215.9 ± 52.1	200.7 ± 45.0***	211.7 ± 51.5	199.6 ± 43.9***
Cit	30.3 ± 6.4	31.3 ± 7.8	30.6 ± 6.5	30.0 ± 6.5	30.7 ± 6.5	30.6 ± 6.6	30.4 ± 6.4	30.7 ± 7.3
Arg	90.2 ± 16.6	93.5 ± 16.9*	90.3 ± 17.0	93.4 ± 15.8**	90.0 ± 17.2	92.7 ± 16.1*	90.6 ± 17.0	91.2 ± 16.2
Gln	558.5 ± 65.5	571.3 ± 62.3	559.6 ± 65.3	562.2 ± 68.3	558.9 ± 64.6	561.7 ± 64.5	560.8 ± 65.4	555.4 ± 68.2
Ser	111.0 ± 18.5	107.6 ± 16.7***	111.8 ± 18.6	107.0 ± 16.0***	113.5 ± 18.6	108.8 ± 17.2***	111.9 ± 18.4	108.8 ± 18.2*
Asn	45.3 ± 6.7	45.6 ± 6.7	45.3 ± 6.8	45.8 ± 6.3	45.4 ± 6.8	45.4 ± 6.4	45.4 ± 6.7	45.4 ± 6.8
Thr	120.9 ± 24.8	122.0 ± 22.6	120.4 ± 24.9	125.6 ± 23.6***	120.9 ± 25.1	122.7 ± 23.4	120.9 ± 25.0	121.9 ± 24.7
His	80.5 ± 9.5	82.1 ± 9.5	79.9 ± 9.2	83.4 ± 9.8***	79.5 ± 9.0	81.8 ± 10.3***	80.3 ± 9.3	81.8 ± 9.7**
Orn	50.7 ± 12.2	55.5 ± 12.1***	50.8 ± 12.6	54.3 ± 11.4***	50.3 ± 12.7	52.7 ± 11.2***	51.1 ± 12.7	52.8 ± 12.0**
Lys	185.9 ± 29.4	197.6 ± 28.1***	185.1 ± 29.6	197.2 ± 27.1***	183.1 ± 29.5	193.1 ± 29.1***	186.4 ± 30.0	192.1 ± 30.0***
Phe	57.4 ± 8.1	62.1 ± 9.5***	56.9 ± 8.1	62.0 ± 8.1***	56.5 ± 8.1	59.3 ± 8.2***	57.4 ± 8.3	60.0 ± 8.5***
Met	25.4 ± 4.2	27.0 ± 4.2***	25.2 ± 4.2	27.5 ± 4.3***	25.0 ± 4.1	26.3 ± 4.3***	25.4 ± 4.3	26.5 ± 4.5***
Pro	135.9 ± 42.4	153.4 ± 47.2***	133.2 ± 41.2	155.2 ± 42.9***	129.7 ± 41.3	144.7 ± 38.7***	135.9 ± 42.9	145.9 ± 40.4***
Trp	57.5 ± 8.9	60.5 ± 11.1***	56.8 ± 8.7	61.2 ± 9.4***	55.9 ± 8.3	59.0 ± 9.1***	57.4 ± 8.8	59.5 ± 10.1***
Ala	338.4 ± 72.4	371.7 ± 68.2***	332.2 ± 69.9	379.2 ± 72.1***	324.6 ± 68.7	359.5 ± 69.9***	337.6 ± 72.3	361.2 ± 75.6***
Tyr	62.4 ± 11.2	69.5 ± 14.0***	61.5 ± 10.9	70.9 ± 12.5***	61.0 ± 11.2	65.8 ± 12.0***	62.4 ± 11.5	66.6 ± 12.5***
Val	216.5 ± 39.7	240.2 ± 39.1***	213.4 ± 38.8	245.9 ± 35.1***	209.2 ± 38.1	229.6 ± 38.9***	216.9 ± 40.9	232.0 ± 40.1***
Leu	118.1 ± 23.4	133.4 ± 22.9***	115.9 ± 22.7	136.9 ± 20.6***	112.9 ± 22.1	126.3 ± 21.9***	118.3 ± 24.1	127.8 ± 23.9***
Ile	59.6 ± 13.7	67.9 ± 13.6***	58.4 ± 13.2	70.5 ± 12.3***	56.6 ± 12.6	64.4 ± 12.9***	59.7 ± 14.1	65.6 ± 14.2***
PFAA index 1	109.7 ± 34.7	136.6 ± 33.6***	106.0 ± 33.0	142.9 ± 28.5***	101.8 ± 32.8	125.0 ± 32.6***	109.6 ± 35.7	127.2 ± 35.5***
PFAA index 2	36.8 ± 14.6	48.2 ± 16.0***	35.5 ± 14.0	49.7 ± 13.4***	33.8 ± 13.6	42.3 ± 14.9***	36.9 ± 15.0	43.7 ± 15.9***

From 2,984 subjects in the cohort study, the subjects without DM (2,729 subjects), the subjects without metabolic syndrome (2,695 subjects), the subjects without dyslipidemia (2,336 subjects), and the subjects without hypertension (2,637 subjects) were included in the follow-up study, respectively. The basal plasma free amino acid concentrations are depicted according to whether the subjects newly developed respective disease or not within the four year follow-up period. Plasma free amino acid concentrations (μmol/L) and index values are shown as means ± standard deviations. Significant differences between Non-developed and Developed subjects are shown as *p < 0.05, **p < 0.01, and ***p < 0.001, respectively.

**Table 3:** The odds ratios for developing new-onset DM, metabolic syndrome, dyslipidemia, or hypertension within the four-year period adjusting for age and gender.

	PFAA index 1	p value	PFAA index 2	p value
The index as a continuous variable Per SD increment				
DM	**2.06 (1.70**–**2.50)**	**<.0001**	**1.77 (1.50**–**2.10)**	**<.0001**
Metabolic syndrome	**3.04 (2.57**–**3.63)**	**<.0001**	**2.27 (1.96**–**2.64)**	**<.0001**
Dyslipidemia	**1.98 (1.74**–**2.26)**	**<.0001**	**1.74 (1.54**–**1.97)**	**<.0001**
Hypertension	**1.42 (1.25**–**1.61)**	**<.0001**	**1.31 (1.17**–**1.47)**	**<.0001**
The index as a categorical variableDM				
First quartile	1.00 (referent)		1.00 (referent)	
Second quartile	**2.39 (1.18**–**5.25)**	**0.0150**	**2.08 (1.08**–**4.28)**	**0.0281**
Third quartile	**4.45 (2.26**–**9.62)**	**<.0001**	**2.99 (1.59**–**6.06)**	**0.0004**
Fourth quartile	**6.85 (3.48**–**14.87)**	**<.0001**	**4.96 (2.66**–**10.01)**	**<.0001**
Metabolic syndrome				
First quartile	1.00 (referent)		1.00 (referent)	
Second quartile	**3.37 (1.55**–**8.40)**	**0.0014**	**4.18 (2.12**–**9.23)**	**<.0001**
Third quartile	**9.31 (4.50**–**22.61)**	**<.0001**	**6.95 (3.61**–**15.11)**	**<.0001**
Fourth quartile	**19.70 (9.56**–**47.75)**	**<.0001**	**14.58 (7.62**–**31.58)**	**<.0001**
Dyslipidemia				
First quartile	1.00 (referent)		1.00 (referent)	
Second quartile	**2.16 (1.55**–**3.02)**	**<.0001**	**1.72 (1.25**–**2.37)**	**0.0009**
Third quartile	**3.03 (2.16**–**4.30)**	**<.0001**	**2.70 (1.96**–**3.75)**	**<.0001**
Fourth quartile	**5.25 (3.66**–**7.60)**	**<.0001**	**4.07 (2.89**–**5.78)**	**<.0001**
Hypertension				
First quartile	1.00 (referent)		1.00 (referent)	
Second quartile	1.01 (0.70–1.47)	0.9476	1.34 (0.94–1.92)	0.1062
Third quartile	**1.90 (1.33**–**2.73)**	**0.0004**	**1.71 (1.21**–**2.44)**	**0.0025**
Fourth quartile	**2.29 (1.60**–**3.32)**	**<.0001**	**2.15 (1.51**–**3.08)**	**<.0001**

Values are odds ratios (95% confidence intervals) for developing these diseases from logistic regressions adjusting for age and gender. Significant odds ratios are highlighted in bold type.

**Table 4:** The odds ratios for developing new-onset DM, metabolic syndrome, dyslipidemia, or hypertension within the four-year period adjusting for age, gender, BMI, FPG, and HOMA-IR.

	DM		Metabolic syndrome		Dyslipidemia		Hypertension	
	Odds ratio	p-value	Odds ratio	p-value	Odds ratio	p-value	Odds ratio	p-value
**Ile**	**1.41 (1.12–1.76)**	**0.003**	**1.30 (1.09–1.55)**	**0.004**	**1.42 (1.22–1.64)**	**<.0001**	1.01 (0.88**–**1.17)	0.838
**Leu**	**1.52 (1.20–1.93)**	**0.001**	**1.34 (1.12–1.61)**	**0.002**	**1.38 (1.19–1.61)**	**<.0001**	0.97 (0.83**–**1.12)	0.653
**Val**	1.24 (0.99**–**1.55)	0.056	1.13 (0.94**–**1.34)	0.191	**1.21 (1.05–1.39)**	**0.007**	0.94 (0.82**–**1.08)	0.388
**Tyr**	**1.23 (1.01–1.49)**	**0.039**	**1.26 (1.08–1.47)**	**0.003**	**1.15 (1.02–1.29)**	**0.024**	1.01 (0.89**–**1.14)	0.893
**Phe**	**1.30 (1.09–1.55)**	**0.004**	1.08 (0.93**–**1.24)	0.326	1.07 (0.95**–**1.20)	0.289	0.99 (0.88**–**1.12)	0.908
**Ala**	1.03 (0.84**–**1.25)	0.796	**1.19 (1.02–1.38)**	**0.028**	**1.35 (1.20–1.51)**	**<.0001**	1.05 (0.93**–**1.19)	0.395
**Pro**	1.09 (0.91**–**1.29)	0.333	1.10 (0.95**–**1.27)	0.199	**1.13 (1.01–1.27)**	**0.026**	0.98 (0.87**–**1.11)	0.798
**Met**	1.18 (0.98**–**1.41)	0.079	1.07 (0.92**–**1.23)	0.385	1.02 (0.91**–**1.15)	0.707	1.00 (0.89**–**1.13)	0.977
**Ser**	1.03 (0.85**–**1.25)	0.759	**0.85 (0.72–0.99)**	**0.039**	**0.80 (0.72–0.90)**	**0.0001**	0.93 (0.83**–**1.04)	0.197
**Gly**	1.03 (0.82**–**1.27)	0.805	0.83 (0.67**–**1.01)	0.071	**0.85 (0.75–0.96)**	**0.009**	0.95 (0.84**–**1.08)	0.464
**PFAA index 1**	**1.31 (1.02–1.69)**	**0.036**	**1.59 (1.29–1.96)**	**<.0001**	**1.72 (1.47–2.02)**	**<.0001**	1.07 (0.91**–**1.25)	0.397
**PFAA index 2**	**1.39 (1.12–1.74)**	**0.003**	**1.36 (1.14–1.63)**	**0.001**	**1.47 (1.28–1.69)**	**<.0001**	1.03 (0.90**–**1.19)	0.641

Values are odds ratios (95% confidence intervals) as a continuous variable per SD increment for developing metabolic syndrome, DM, dyslipidemia, or hypertension from logistic regressions. Significant odds ratios are highlighted in bold type.

**Table 5:** The odds ratios for developing new-onset DM, metabolic syndrome, or dyslipidemia within the four-year period adjusting for age, gender, BMI, FPG, HOMA-IR, waist circumference, DBP, SBP, TG, HDL-C, LDL-C, and T-CHO.

	DM		Metabolic syndrome		Dyslipidemia	
	Odds ratio	p-value	Odds ratio	p-value	Odds ratio	p-value
**PFAA index 1**	1.24 (0.95–1.62)	0.114	**1.41 (1.12–1.76)**	**0.003**	**1.34 (1.12–1.60)**	**0.001**
**PFAA index 2**	**1.30 (1.04–1.63)**	**0.021**	**1.26 (1.04–1.53)**	**0.017**	**1.20 (1.02–1.40)**	**0.028**

Values are odds ratios (95% confidence intervals) as a continuous variable per SD increment for developing metabolic syndrome, DM, or dyslipidemia from logistic regressions. Significant odds ratios are highlighted in bold type.
