# Supplementary material for: Plasma Free Amino Acid Profiles Predict Four-Year Risk of Developing Diabetes, Metabolic Syndrome, Dyslipidemia, and Hypertension in Japanese Population
Source: Sci Rep. 2015 Jul 9;5:11918. doi: 10.1038/srep11918 (PMC4496670; doi:10.1038/srep11918)
Supplement: Supplementary Table S1 [file srep11918-s1.doc]

SUPPLEMENTARY INFORMATION

**Plasma Free Amino Acid Profiles Predict Four-Year Risk of Developing Diabetes, Metabolic Syndrome, Dyslipidemia, and Hypertension in Japanese Population**

Minoru Yamakado, Kenji Nagao, Akira Imaizumi, Mizuki Tani, Akiko Toda, Takayuki Tanaka, Hiroko Jinzu, Hiroshi Miyano, Hiroshi Yamamoto, Takashi Daimon, Katsuhisa Horimoto, and Yuko Ishizaka

**Supplementary Table S1**

The plasma free amino acid concentrations of healthy subjects, patients of DM, metabolic syndrome, dyslipidemia, and hypertension at the beginning of the cohort study

|  | Healthy subjects | DM | Metabolic syndrome | Dyslipidemia | Hypertension |
| --- | --- | --- | --- | --- | --- |
| N  (male, female) | 1,962  (1,033, 929) | 255  (211, 44) | 289  (275, 14) | 648  (572, 76) | 347  (271, 76) |
| Gly | 216.0 ± 52.6 | 184.6 ± 32.4*** | 181.3 ± 29.8*** | 190.3 ± 39.1*** | 192.5 ± 37.3*** |
| Cit | 30.5 ± 6.5 | 31.4 ± 7.1 | 29.6 ± 6.5 | 29.9 ± 6.6 | 30.5 ± 6.4 |
| Arg | 90.1 ± 17.1 | 93.4 ± 17.1* | 91.3 ± 15.1 | 91.2 ± 15.6 | 90.9 ± 15.7 |
| Gln | 559.9 ± 65.4 | 554.1 ± 67.0 | 548.9 ± 63.1* | 556.4 ± 68.7 | 550.9 ± 62.2 |
| Ser | 113.0 ± 18.6 | 110.1 ± 17.8 | 105.3 ± 17.5*** | 104.4 ± 16.7*** | 105.6 ± 17.1*** |
| Asn | 45.5 ± 6.6 | 44.8 ± 6.7 | 44.6 ± 6.1 | 44.8 ± 6.7 | 44.6 ± 6.6 |
| Thr | 121.1 ± 25.2 | 124.0 ± 26.0 | 123.5 ± 24.4 | 121.0 ± 24.8 | 122.3 ± 23.7 |
| His | 79.8 ± 9.0 | 81.8 ± 10.3** | 84.2 ± 11.1*** | 83.1 ± 9.9*** | 81.5 ± 10.8** |
| Orn | 50.2 ± 12.3 | 57.2 ± 13.9*** | 55.2 ± 12.2*** | 54.3 ± 12.6*** | 53.1 ± 12.2*** |
| Lys | 183.9 ± 29.8 | 199.1 ± 30.1*** | 199.1 ± 29.0*** | 196.4 ± 28.0*** | 190.7 ± 26.5*** |
| Phe | 56.7 ± 8.0 | 61.9 ± 9.6*** | 63.1 ± 8.5*** | 61.4 ± 8.6*** | 59.7 ± 8.7*** |
| Met | 25.2 ± 4.2 | 27.1 ± 4.8*** | 27.7 ± 4.2*** | 26.9 ± 4.3*** | 26.1 ± 4.2*** |
| Pro | 131.5 ± 40.9 | 153.6 ± 41.1*** | 164.3 ± 43.8*** | 158.2 ± 43.4*** | 145.3 ± 44.7*** |
| Trp | 56.5 ± 8.5 | 58.8 ± 9.3*** | 62.9 ± 9.7*** | 62.3 ± 9.6*** | 58.6 ± 9.7*** |
| Ala | 328.4 ± 69.3 | 380.3 ± 78.4*** | 403.7 ± 67.5*** | 386.2 ± 70.6*** | 362.9 ± 75.4*** |
| Tyr | 61.2 ± 11.2 | 70.2 ± 13.2*** | 72.5 ± 11.5*** | 68.8 ± 11.6*** | 66.9 ± 12.0*** |
| Val | 210.6 ± 38.4 | 249.2 ± 42.9*** | 254.7 ± 39.6*** | 246.2 ± 38.2*** | 230.9 ± 40.5*** |
| Leu | 114.2 ± 22.4 | 137.4 ± 25.1*** | 142.3 ± 22.3*** | 137.8 ± 22.1*** | 126.5 ± 24.0*** |
| ILE | 57.3 ± 12.8 | 72.1 ± 15.1*** | 74.7 ± 14.0*** | 71.8 ± 13.9*** | 65.0 ± 14.5*** |
| PFAA index 1 | 103.0 ± 32.7 | 148.0 ± 34.6*** | 155.8 ± 29.7*** | 142.4 ± 31.8*** | 131.0 ± 35.9*** |
| PFAA index 2 | 34.3 ± 13.8 | 51.6 ± 16.6*** | 54.8 ± 15.7*** | 50.1 ± 14.7*** | 44.6 ± 16.2*** |

Plasma free amino acid concentrations (μmol/L) and index values are shown as means ± standard deviations. Statistical differences were analyzed by one-way analysis of variance (ANOVA) with Dunnett's multiple comparison test. Significant differences between healthy subjects and patients of DM, metabolic syndrome, dyslipidemia, or hypertension are shown as *p<0.05, **p<0.01, and ***p<0.001, respectively. In the total of 2,984 subjects who enrolled in the four year cohort study, each category is defined as follows. Healthy subjects include those without DM, metabolic syndrome, dyslipidemia, or hypertension at the beginning of the study. DM, Metabolic syndrome, Dyslipidemia, or Hypertension include those subjects that had one or more of these conditions at the beginning of the study.
