# Supplementary material for: Plasma Free Amino Acid Profiles Predict Four-Year Risk of Developing Diabetes, Metabolic Syndrome, Dyslipidemia, and Hypertension in Japanese Population
Source: Sci Rep. 2015 Jul 9;5:11918. doi: 10.1038/srep11918 (PMC4496670; doi:10.1038/srep11918)
Supplement: Supplementary Figure S1 [file srep11918-s2.pdf]

Plasma Free Amino Acid Profiles Predict Four-Year Risk of Developing Diabetes, Metabolic Syndrome, Dyslipidemia, and Hypertension in Japanese Population

Minoru Yamakado, Kenji Nagao, Akira Imaizumi, Mizuki Tani, Akiko Toda, Takayuki Tanaka,
Hiroko Jinzu, Hiroshi Miyano, Hiroshi Yamamoto, Takashi Daimon, Katsuhisa Horimoto, Yuko Ishizaka

	Ile	Leu	Val	Tyr	Phe	Ala	Pro	Met	Lys	Trp	Orn	His	Thr	Arg	Gln	Cit	Asn	Ser	Gly
VFA	29	28	49	97	6	90	20	6	5	49	6	8	5	6	18	5	56	15	94
Ins120min	100	16	0	91	54	41	0	42	0	61	0	44	8	10	1	0	44	37	45

Supplementary Figure S1.

Frequencies of amino acids that were selected from the top 100 models for each objective variable

The numbers indicate the percentages of each amino acid selected for generating the multiple linear regression analysis with variable selection against each objective variable (VFA or Ins120min values).
